# Efficient conditioned pain modulation despite pain persistence in painful diabetic neuropathy

**DOI:** 10.1097/PR9.0000000000000592

**Published:** 2017-04-20

**Authors:** Yelena Granovsky, Hadas Nahman-Averbuch, Mogher Khamaisi, Michal Granot

**Affiliations:** a The Laboratory of Clinical Neurophysiology, Technion Faculty of Medicine, Haifa, Israel; b Faculty of Health and Welfare Sciences, University of Haifa, Haifa, Israel; c Internal Medicine D and Institute of Endocrinology, Diabetes and Metabolism, Rambam Health Care Campus, Technion Faculty of Medicine, Haifa, Israel; d Department of Neurology, Rambam Health Care Campus, Haifa, Israel

**Keywords:** Painful diabetic neuropathy, Pain modulation, Pain duration, Pain intensity, Conditioned pain modulation, Temporal summation

## Abstract

**Introduction:**

Alleviation of pain, by either medical or surgical therapy, is accompanied by transition from less efficient, or pro-nociceptive, to efficient conditioned pain modulation (CPM). Spontaneous decrease or resolution of pain with disease progression is reported for some patients with painful diabetic neuropathy (PDN).

**Objectives:**

To explore whether CPM changes similarly in parallel to spontaneous resolution of pain in PDN patients.

**Methods:**

In this cross-sectional study, thirty-three patients with PDN underwent psychophysical assessment of pain modulation on the forearm, remote from the clinical pain.

**Results:**

Pain duration was not correlated with neuropathic pain intensity, yet, it correlated with CPM efficiency; patients with longer pain duration had same pain level, but more efficient CPM than those with short-pain duration (ρ = −0.417; *P* = 0.025, Spearman correlation). Patients with pain more than 2 years (median split) expressed efficient CPM that was not different from that of healthy controls. These patients also had lower temporal summation of pain than the short-pain duration patients group (*P* < 0.05). The 2 patient groups did not differ in clinical pain characteristics or use of analgesics.

**Conclusion:**

Pro-nociception, expressed by less efficient CPM and high temporal summation that usually accompanies clinical painful conditions, seems to “normalize” with chronicity of the pain syndrome. This is despite continuing pain, suggesting that pro-nociceptivity in pain syndromes is multifactorial. Because the pain modulation profile affects success of therapy, this suggests that different drugs might express different efficacy pending on duration of the pain in patients with PDN.

## Abstract 2

Despite persistent clinical pain in patients with painful diabetic neuropathy, longer pain duration was associated with more efficient CPM.

## 1. Introduction

Neuropathic pain is a common complication of diabetes characterized by burning pain on the affected limbs. Similar to other pain states, patients with painful diabetic neuropathy (PDN) demonstrate enhanced activity of facilitatory pain pathways^26^ and deficient activity of pain inhibitory pathways,^25^ pointing thus to a pro-nociceptive pattern of central pain modulation.

An interesting feature of the neuropathic pain of diabetic origin relates to the reports of spontaneous pain improvement or resolution with disease progression.^3,4,40^ Studies on surgical and medication treatments for pain have shown that the pain alleviation is accompanied by improvement of pro-nociceptivity, with transition from less efficient to efficient conditioned pain modulation (CPM).^6,13,18,25,49^ However, it is unknown whether the spontaneous alleviation of neuropathic pain along with PDN duration is similarly associated with reversal of pro-nociceptivity.

The aim of this study was to evaluate pain modulation in PDN of different pain duration and its association with pain levels. We hypothesized that longer neuropathic pain duration would be associated with lower clinical pain levels and reversal of the pro-nociceptive characteristics of pain modulatory system.

## 2. Methods

### 2.1. Subjects

Thirty-five patients with PDN participated in this study. The diagnosis of PDN was confirmed by a qualified neurologist or endocrinologist with special training in diabetes care. This article reports the results of baseline (pretreatment) assessment of the patients cohort who then underwent treatment with duloxetine (reported at Yarnitsky et al, 2012^49^). Patients met the following inclusion criteria: (1) lower limb distal neuropathic pain for more than 3 months and (2) mean pain severity during the last month of ≥40 on a 0 (“no pain at all”) to 100 (“most painful imaginable sensation”) numerical pain scale (NPS). The exclusion criteria were as follows: (1) intake of a monoamine oxidase inhibitor (MAO-I) within the last 14 days, (2) narrow-angle glaucoma, (3) inability to undergo psychophysical testing, (4) pain complaints at the upper limbs, and (5) chemotherapy for cancer and known vitamin B12 deficiency. Patients who were on regular use of serotonin–noradrenaline reuptake inhibitors (SNRIs) were tested after 1 month drug washout.

Data from healthy subjects (N = 29, 17 women) collected under same psychophysical protocol as for patients with PDN served as the control group. The inclusion criteria for the control group were as follows: (1) absence of chronic pain history; (2) no use of analgesic or psychiatric medication on a regular basis; (3) age between 40 and 80. All participants were able to communicate and understand the instructions of the study and were asked to refrain from any pain relief medications 24 hours before the experimental trial. The study was approved by the local ethics committee, and informed consent was obtained from all participants.

### 2.2. Experimental protocol

All experiments were conducted in the same setting by the same experimenter (H.N.-A.) at the Laboratory of Clinical Neurophysiology in Rambam Health Care Campus, Haifa, Israel. Subjects sat comfortably in a quiet room with an ambient room temperature of 22°C to 23°C.

#### 2.2.1. Clinical assessment

Patients were asked to report duration of (1) diabetes, (2) sensory neuropathic symptoms such as, (3) mean and maximal neuropathic pain intensity during the last week, and pain duration. In addition, the information regarding current or past pain-relieving drugs consumption was collected.

As part of a clinical examination, sensory detection thresholds were measured at the dorsum of the foot to assess neuropathy severity. Warm detection thresholds (WDT) and cold detection thresholds (CDT) were measured 3 times each using the thermal sensory analyzer (TSA-II) (Medoc, Ramat Yishai, Israel) with a Peltier 30 × 30 mm^2^ contact thermode according to the method of limits.^45^ The 32°C baseline temperature decreased or increased at a rate of 1.0°C/s and was stopped by pressing the response button at the moment that warm or cold sensation was perceived. Warm detection threshold and CDT were calculated as the mean values of the 3 measurements. The mechanical detection thresholds (MDT) were measured using von Frey filaments (North Coast Medical, San Jose, CA). The weight was increased using consecutive filaments until the patient reported that sensation was perceived. Each filament was applied 3 times until the filament pressure was detected in at least 2 of the 3 trials. The first filament to be detected at ≥2/3 times was determined as the MDT.

#### 2.2.2. Psychophysical assessment

All participants, the patients with PDN and the control subjects, underwent CPM and temporal summation (TS) assessments to assess pain modulation. All tests were delivered to the volar aspect of the forearm. This site is less commonly affected by the neuropathy, allowing normal or near-normal peripheral sensory function. This is because genuine assessment of the central effect depends on intactness of the peripheral conduction of neural signals.

Mechanical temporal summation of pain (TS)—the stimuli were delivered with an 180gr (# 6.45) von Frey filament applied to the volar forearm. Subjects were exposed to a series of 10 stimuli with an interstimulus interval of 1 sec, applied within an area of 1 cm in diameter. Subjects were asked to rate the level of pinprick pain intensity using verbal NPS for the first and the tenth stimuli. The difference between the tenth and the first pain scores served as a score of the TS.

Conditioned pain modulation was assessed using the parallel paradigm in which 2 identical test stimuli were given; once before and then simultaneously with a noxious conditioning stimulus. The test stimulus was a tonic noxious contact heat stimulus applied to the volar aspect of the dominant forearm using TSA. The intensity of the test stimulus was predetermined individually based on the psychophysical parameter of Pain60 temperature. This method was based on delivery of several triplets of 7-sec long stimuli of various intensities; the closest temperature that induced pain at a level of 60 on a 0-100 NPS was considered as the Pain60 temperature.^10^ The baseline temperature was 32°C, which increased at a rate of 2°C/s to the destination temperature, which was held for 30 seconds, and decreased back to baseline at the same rate. Subjects were asked to rate the intensity of the test stimulus along the stimulus at the 10th, 20th, and 30th second. The mean pain score served as the pain level of “test stimulus.” After a 15-minute break, subjects were exposed to the conditioning stimulus by immersion of the nondominant hand into a hot water bath at 46.5°C (Heto CBN 8-30 Lab equipment, Heto-Holten A/S; Allerod, Denmark) up to the wrist for 1 minute. Water-induced pain was rated every 10 seconds during the first 30 seconds of immersion; the mean score of the 3 ratings of the immersed hand served as the pain level of the “conditioning stimulus.” During the last 30 seconds of the conditioning stimulation, an identical test stimulus was repeated, and pain of the test stimulus was rated again every 10 seconds. The CPM effect was calculated as the difference between 2 test-stimuli applications: the conditioned test stimulus minus the stand-alone test stimulus, for each single individual pair of stimuli, obtained at 10, 20, and 30 seconds of test stimuli (CPM_10_, CPM_20_, and CPM_30_, respectively), and as CPM_mean_ for averaged 3 CPM values. More negative values indicated more efficient CPM. This paradigm of conditioned pain stimulation is routinely used in our laboratory.^10,23,24,43^

#### 2.2.3. Questionnaires

At the beginning of the experimental session, all participants completed several pain-related psychometric questionnaires for the assessment of pain catastrophizing (assessed with Pain Catastrophizing Scale [PCS])^35^ and anxiety levels (assessed with Spielberger State-Trait Anxiety Inventory, STAI).^34^ The patients with PDN also reported their self-perceived health by filling out a Hebrew version of the short-form 36 (SF-36) Health Survey.^20^ SF-36 contains the following subscales: vitality, physical functioning, bodily pain, general health perceptions, physical role functioning, emotional role functioning, and social role functioning. Higher scores on each summary scale agree with better general health.

### 2.3. Statistical analysis

The primary interest of this study is to characterize the relationship between pain duration and pain psychophysics. Preliminary examination of data indicated that skewed pain duration data distributions, with some extreme values, would result in problematic Pearson correlations, with extreme points having unacceptably high leverage. Therefore, nonparametric Spearman (rank-order) correlations were performed to test overall monotonicity of relationships between neuropathic pain level and duration and psychophysical measures. To further examine the effect of pain duration on pain modulation profile (PMP) characteristics, additional analyses were conducted by dividing the patients into 2 groups based on the median score of pain duration. The results of 2 subgroups of patients were compared with the control subjects with the addition of age as a covariate, in a regression model. The post hoc analysis was performed to confirm the group differences. The level of significance was set at *P* < 0.05. The data are presented as mean (SE) if not indicated otherwise. Statistical analyses were performed using Microsoft Excel (Microsoft Corporation, Redmond, WA) and JMP (SAS Institute, Cary, NC).

## 3. Results

Two patients did not provide full information about their neuropathic pain duration, so the final sample of patients with PDN consisted of N = 33 (7 females). Demographic and clinical parameters as well as the values of PCS and STAI of the patients with PDN as well as the results of sensory quantitative sensory assessment are presented in Table 1. Patients were significantly older than controls (60.4 ± 9.7 vs 53.9 ± 6.7 years, mean ± SD, *P* = 0.004); therefore, subjects' age was added as a covariate into the regression models.

**Table 1:**
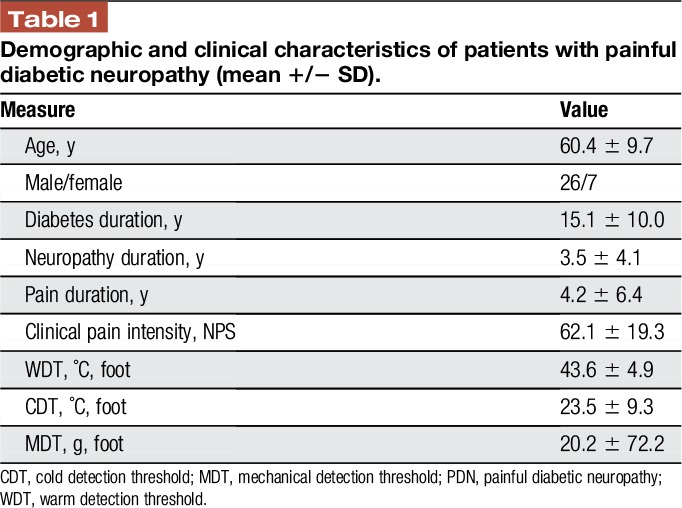
Demographic and clinical characteristics of patients with painful diabetic neuropathy (mean +/− SD).

### 3.1. Pain duration and neuropathy severity

Patients with longer pain duration had (1) higher duration of neuropathy (ρ = 0.826; *P* < 0.001); (2) higher severity of the neuropathy as measured by higher CDT (ρ = −0.366; *P* = 0.036) and warm detection threshold (ρ = 0.350; *P* = 0.046), all tested on feet. Because no consistent responses were obtained to the assessment of mechanical detection threshold, these data are not reported. It is important that the pain duration did not correlate with neuropathic pain intensity (ρ = −0.154; *P* = 0.393) or age (ρ = 0.116; *P* = 0.522).

### 3.2. Pain modulation and neuropathic pain intensity and duration

**Figure 1.**
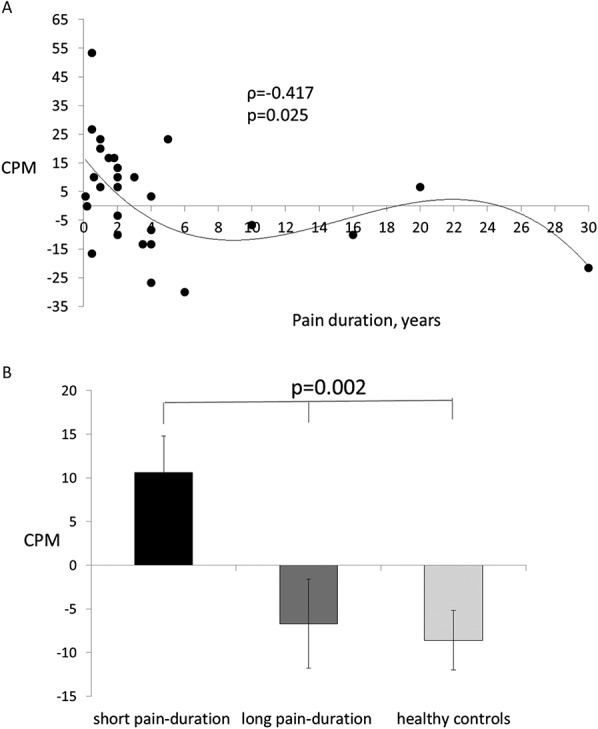
(A) Neuropathic pain duration significantly correlated with CPM efficiency, Spearman ρ = −0.417; *P* = 0.025. The solid line is a spline fit to show the general monotonicity of the relationship, lambda = 100. (B) PDN patients with longer pain duration had more efficient CPM than the short-pain duration patients (*P* < 0.05), and were not different from the responses from healthy controls. CPM, conditioned pain modulation; PDN, painful diabetic neuropathy.

No significant correlations were found between the neuropathic pain intensity and CPM_mean_ (ρ = 0.206; *P* = 0.285) or its temporal segments; CPM_10_ (ρ = 0.199, *P* = 0.302), and CPM_20_ (ρ = 0.292, *P* = 0.132) or CPM_30_ (ρ = 0.130, *P* = 0.517). In line, no correlation was found between pain intensity and TS (ρ = 0.051, *P* = 0.783). However, looking at the relationships between pain duration and pain modulation capabilities, we found that patients with longer pain duration had (1) increased efficiency of CPM_mean_ (ρ = −0.417; *P* = 0.025, Fig. 1A); when analyzed by segments along the CPM test, the association between the pain duration and CPM was significant for CPM_10_ (ρ = −0.625, *P* < 0.001) and CPM_20_ (ρ = −0.457, *P* = 0.015) but not for CPM_30_ (ρ = −0.120, *P* = 0.551), (2) a trend toward lower TS magnitude (ρ = −0.316, *P* = 0.079).

### 3.3. Pain modulation in patients grouped by pain duration vs control subjects

Patients were divided into 2 groups based on the median split of the pain duration. Patients with pain duration of 2 years or less (N = 20, mean age 60.6 ± 11.7, mean ± SD) were considered as short-pain duration group, whereas patients with pain duration longer than 2 years (N = 13, mean age 60.2 ± 6.2, mean ± SD) were considered as long-pain duration group. The results of 3 group comparisons for psychophysical variables are presented in Table 2. Patients with longer pain duration demonstrated lower TS magnitude in comparison with the short-pain duration group. Moreover, patients with longer pain duration demonstrated more efficient CPM_mean_ (Fig. 1B) and the CPM at 10 (CPM_10_) and 20 seconds (CPM_20_) of the CPM assessment, as compared with short-pain duration patients, and were not different of those from the healthy controls.

**Table 2:**
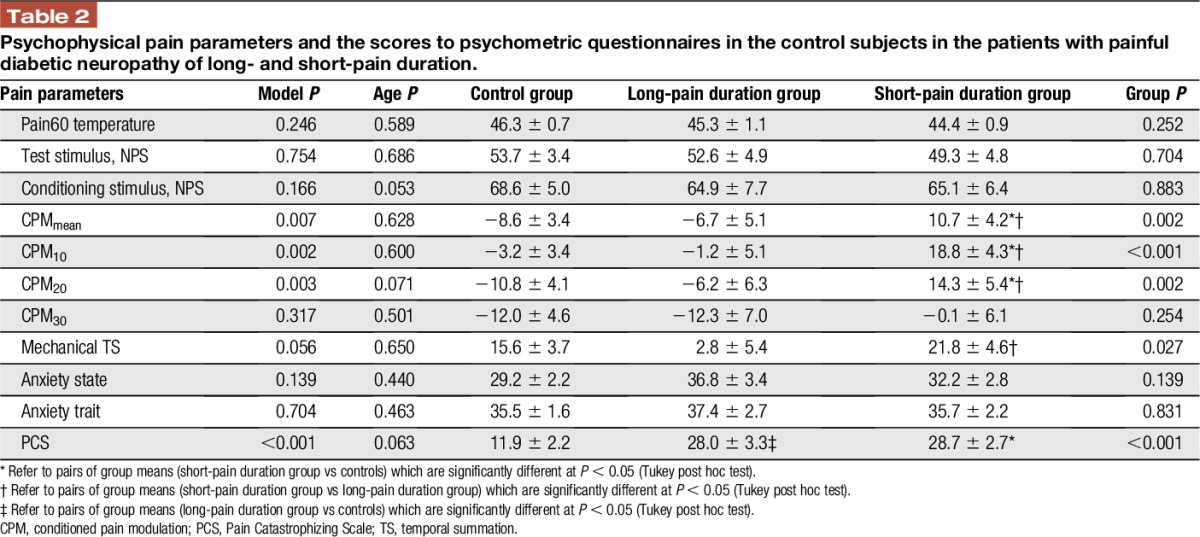
Psychophysical pain parameters and the scores to psychometric questionnaires in the control subjects in the patients with painful diabetic neuropathy of long- and short-pain duration.

### 3.4. Pain-related psychometric variables

No correlation was observed between the pain duration and any of the pain-related psychophysical parameters; anxiety trait (*r* = 0.055; *P* = 0.784), anxiety state (*r* = 0.276; *P* = 0.174), or PCS (*r* = 0.126; *P* = 0.531). Comparing the long- and short-pain duration PDN patients with the healthy subjects, no significant group differences were observed for the scores for anxiety state or trait; the PCS scores were significantly higher in the patients with PDN, with no differences between the 2 patient groups (Table 2).

### 3.5. Clinical characteristics of the short- and long-pain duration painful diabetic neuropathy patient groups

No significant group differences were observed for the clinical pain intensity (63.5 ± 17.4, NPS, in short-pain duration groups vs 61.3 ± 20.9 in long-pain duration group, mean ± SD, *P* = 0.754), or for the neuropathy severity in terms of foot warm (42.3 ± 4.7 vs 45.2 ± 4.9, mean ± SD, *P* = 0.112) or cold sensation threshold (25.8 ± 6.5 vs 19.9 ± 11.9, mean ± SD, *P* = 0.081) (although a trend of increase in severity with time can be seen). In addition, the short- and long-pain duration patients were not different for any component of the SF-36 Health Survey (Table 3).

**Table 3:**
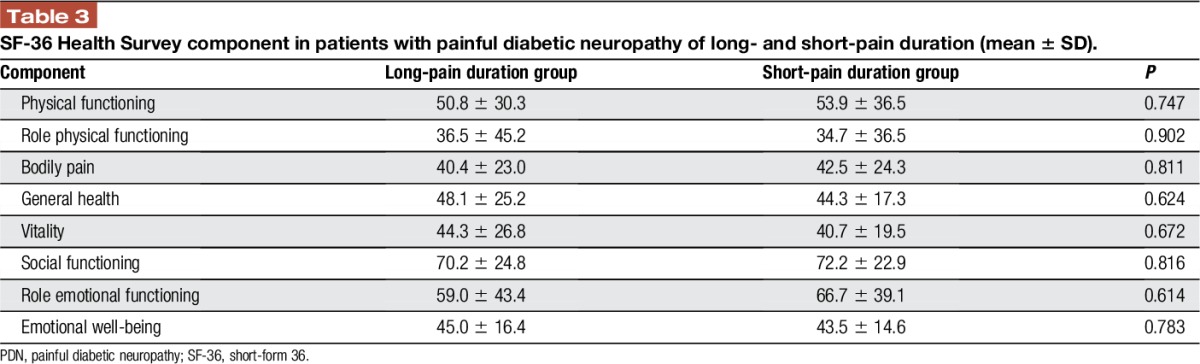
SF-36 Health Survey component in patients with painful diabetic neuropathy of long- and short-pain duration (mean ± SD).

Among the 33 patients, 17 (52%) were currently using pain medications (nonsteroidal anti-inflammatory drugs, sSNRIs, or gabapentinoids). The proportion of patients on pain medications was not different between the short (10 patients with and 10 patients without medications) and long (6 and 7 patients, respectively) pain duration groups (2-tailed Fisher exact test *P* = 1.00). In addition, the number of used drug families was similar between short- and long-pain duration patients with PDN (1.9 ± 0.9 vs 1.5 ± 0.5, mean ± SD, *P* = 0.317).

## 4. Discussion

The main finding of this study is that the association of clinical pain and pronociceptive pain modulation is not maintained in long-standing PDN. Although we did not find longer pain duration to be associated with lower pain intensities, it was, however, associated with more efficient CPM response, as well as less enhanced TS. In other words, although clinical pain levels seem to persist, CPM and TS seem to “return to normal” along the neuropathic pain syndrome. The longer pain-efficient CPM association was unrelated to general functioning, physical or emotional health or to analgesics intake.

We have advocated the concept of PMP, which reflects the state of individual pain modulation in various clinical situations, showing the shifts in modulation in accordance with changes in clinical pain.^46,48^ A central point is that the presence of clinical pain is usually accompanied by a shift toward pro-nociception, expressed, among other parameters, by a less efficient CPM and enhanced TS. This has been shown for various pain syndromes, including neuropathic pain.^21,39,46^ In line, the association between longer pain duration and pro-nociceptive PMP was reported for some nonneuropathic pain states such as osteoarthritis.^2,32^ It is important that subsequent to the pro-nociception reported during pain, evidence is available to show that a shift back toward normal PMP occurs when pain is alleviated after surgical^13,18^ or pharmacological treatment.^25,49^ However, efficient engagement of descending inhibition and decreased activity in pain facilitatory pathways provide protection against the development of experimental chronic neuropathic pain models in animals,^8,29^ and predict lower incidence and intensity of chronic postoperative pain in humans.^44,47^ Thus, a relationship between clinical pain and pro-nociceptive pain modulation was established; however, the “chicken-egg” question remains open.

It should be emphasized that our study is cross sectional, so we cannot definitively comment on “normalization” of the modulation along time in our patients without a preplanned long-length prospective study, but can extend this concept from our findings that CPM in long-pain duration patients was not different from the CPM responses in healthy subjects. We hypothesize, therefore, that better CPM in patients with long-pain duration may represent a recovery of the modulation in a “back to normal” fashion. There seems to be an effort of the central nervous system pain-controlling subsystems to return to equilibrium, even when the perturbing factor, pain, is still prevalent. A similar situation is proposed regarding reduced TS magnitude in patients with longer duration of neuropathic pain.

Neuropathic pain is a result of excessive primary afferent activity that generates neuronal hyperexcitability in second-order spinal neurons and modulates their pattern of functioning.^15^ Alterations of pain modulatory pathways, with a shift toward a pro-nociception, were demonstrated in psychophysical studies in patients with neuropathic pain. Enhanced TS and less-efficient CPM were reported for various neuropathic pain conditions.^1,24,37^ These studies, however, did not explore the relationship between the neuropathic pain duration and pain modulation capabilities. It is worth noting that spontaneous improvement or even partial or complete resolution of spontaneous pain is reported for several studies on patients with PDN.^3,4,22,40^ The likely cause is loss of nociceptive neurons that generate pain along the process of ongoing metabolic and microvascular changes.^16,27^ Our results did not reveal association between longer pain duration and low pain intensity, perhaps because we only recruited patients reporting pain, so our group was biased. Therefore, despite significant correlation between the duration of pain symptoms and duration of the neuropathy, the efficient pain inhibitory capacity in long-pain duration patients seems to be determined by central rather than peripheral changes, suggesting an adaptation process of the pain processing system.

Our findings on antinociceptive shift of PMP with no pain resolution are challenging. We suggest that central neuroplastic changes that took place during the years of diabetic neuropathy might have contributed to the efficiency of pain inhibitory systems. At the initial stage, the net output of descending pain modulation is shifted to overactive pain facilitatory mechanisms, promoting the development and maintenance of persistent neuropathic pain.^30,31^ As was documented by animal neuropathic pain models, inactivation of supraspinal structures involved in descending facilitation prevents or diminishes the evoked secondary hyperalgesia.^17,42^ We may, therefore, suggest that pro-nociceptive features of the PMP in PDN patients with shorter pain duration relate to the superior activity of pain facilitatory pathways. In line, we may propose that neuroplastic alterations occurring at various levels of pain neuromatrix modulate the balance between activity of descending pain facilitatory and inhibitory pathways in favor of the latter. These plastic changes, theoretically, may strengthen the anatomical connectivity within the periaqueductal gray–rostral ventromedial medulla–spinal circuit, and with the subnucleus reticulates dorsalis, a part of the spinal-bulbo-spinal loop implicated in diffuse noxious inhibitory control.^19,41^ This possibly explains why patients with longer pain duration expressed more efficient CPM. In line, functionally active descending inhibition on the second-order neurons may also contribute to lower TS magnitude and higher pain thresholds.

The functional changes in the pain-modulatory system at the brainstem level may involve descending inputs from higher brain structures.^5^ Reports on neuropathic pain, including diabetes, indicate enhanced activation in sensory and limbic pain–related brain structures^28,36^ and changes in cortical thickness.^7,9^ It is important that neuropathic pain duration was positively correlated with increased BOLD signal in some of these structures,^36^ and with enhanced cortical thickness in anterior insula along with thinness in prefrontal cortex.^7^ We, therefore, hypothesize that the proposed anatomical and functional changes in the pain-related brain structures occurring through pain duration might be a part of a general long-term reorganization of the pain-modulatory system. We further suggest that this reorganization is more relevant to sensory rather than to affective components of pain perception because pain duration was not associated with magnitude of pain-related psychometrics such as anxiety or pain catastrophizing.

Our results shed light on 2 additional aspects of potential clinical implication of PMP; first, the significant relationship between pain duration and CPM was restricted to first 20 seconds of dual stimulation (CPM_10_ and CPM_20_), confirming a methodological advantage of shorter CPM assessment.^11^ Second, less-efficient CPM at early stages of painful neuropathy may suggest higher efficacy of mechanism-based analgesic choice (such as serotonin-norepinephrine reuptake inhibitors) during the first years of the neuropathic pain, whereas other treatment choices may be preferable for the long-time pain sufferers. This approach, however, should be confirmed from a study on a large group of patients with PDN.

Our main study limitation is the quite small sample, which is a general problem in clinical research. In addition, the middle-aged control subjects were significantly younger than the patients with PDN. Although age was included as a covariate in all group comparisons, the younger age of control subjects can be a potential bias for the results on CPM because of several reports on more-efficient CPM at younger ages.^12,14^ However, this issue is far from being conclusive because of contrary findings.^33,38^ The other limitation is that this is a cross-sectional study; the ideal design for a study that relates to durations is a longitudinal study. Because no prospective follow-up was designed, we rely on patients' memory as far the durations of both neuropathy and pain, rather than on documented data.

To conclude, this is a first report on the association between duration of neuropathic pain and pain modulation. Pain modulation seems to return to normal for longer duration of pain, whereas clinical pain levels seem to persist for a long time, at least in our cohort of patients with PDN. Our findings on the difference of antinociceptive PMP in PDN patients with longer pain duration highlight the significant role of the “time window” in which the assessment of PMP has clinical meaning and should be considered as a factor in treatment choice and evaluation of treatment efficacy.

## Disclosures

The authors have no conflicts of interest to declare.

This study was sponsored by an IIT grant from Eli Lilly Inc.

Y. Granovsky contributed to logistics of this study, data analysis, and the manuscript preparation. H. Nahman-Averbuch contributed to data collection. M. Khamaisi contributed to the patients recruitment and consenting. M. Granot contributed to data analysis and the manuscript preparation. All the authors approved the final draft of the manuscript.
